# Do Chinese Preschool Children Love Their Motherland? Evidence from the Game-Based Assessment

**DOI:** 10.3390/bs14100959

**Published:** 2024-10-16

**Authors:** Qianqian Liu, Xiumin Hong

**Affiliations:** Faculty of Education, Beijing Normal University, Beijing 100875, China; qianqianliu@bnu.edu.cn

**Keywords:** patriotism, preschool children, game-based assessment

## Abstract

Patriotism is a topic of significant importance in many countries around the world. Preschool children play a crucial role in shaping the future, and their patriotism is closely linked to the future development of their nation. Currently, the game-based assessment has advantages over traditional evaluation methods and is more suitable for preschool children. This study employed a game-based assessment method to investigate patriotism among preschool children aged 3 to 6 years in China. The results indicated that their levels of patriotism were above average and tended to increase with age. Preschool children in the capital region scored higher on national cognitive mastery. However, preschool children’s understanding of patriotism remains somewhat vague, and they often articulate and express their patriotism through concrete examples. Four types of patriotism among preschool children were identified: high-level patriotism, cognitive-based patriotism, emotional-based patriotism, and low-level patriotism. The findings of this study contribute to a more comprehensive understanding of patriotism in preschool children and provide an evidence-based reference for the development of patriotic education.

## 1. Introduction

Patriotism is commonly defined as a citizen’s love for their country, and it also influences the citizen’s willingness to contribute to their nation. Generally speaking, the development of a country relies on various supporting factors, with the patriotism of its people being one of the most significant. Preschool children are in the early stages of individual development, which is a crucial period for fostering the germination of patriotism. China has placed great importance on cultivating individual patriotism since ancient times. However, we believe that few existing studies adequately explore the concept of patriotism among preschool children in China. Therefore, this study aims to identify the primary characteristics of patriotism in Chinese preschool children. Game-based evaluation methods were employed due to their numerous advantages over traditional assessment techniques, particularly for young children. Engaging and enjoyable games enhance preschool children’s participation and involvement, leading to richer and more accurate data while minimizing errors associated with artificial data collection. Ultimately, these game-based assessments provide evidence-based insights for enhancing patriotism among preschool children.

### 1.1. The Importance of Patriotism in Young Children

Patriotism is defined in various ways, including a sense of national loyalty, an appreciation for national symbols, beliefs regarding a country’s superiority, and a vital component in fostering civic ties within a mature nation [1,2]. In light of increasing concerns about a diminished sense of national identity and belonging among citizens, politicians and educators are advocating for schools to cultivate a robust modern sense of patriotism and a shared purpose that can unite individuals and motivate citizens to fulfill their responsibilities to one another and to their country [3,4]. The development of each state is directly influenced by the foundational beliefs instilled in children. The formation of patriotism is unattainable unless it is anchored in personal feelings and a sense of public significance [5]. Patriotism serves as the cornerstone of a nation’s progress; if citizens do not love their country, it risks losing its developmental momentum. The patriotic education of younger generations is a crucial aspect of a state’s educational policy. For instance, traditional approaches to civic education for young children in the United States often emphasize the acquisition of patriotic knowledge, such as recognizing flags and leaders, as well as practicing fundamental civic skills like voting and decision making [6].

In any country, the significance of preschool children in the nation’s development is indisputable. This emerging generation ensures continuity in the advancement and progressive changes within society. Preschool children are not only citizens but also the future architects of their country. From an early age, fundamental values that shape their personalities are instilled in these future citizens, including their attitudes toward their home, their motherland, and their relationships with those around them, including their closest community and their own self-identity within that community. Among these values, attitudes toward the motherland are particularly crucial, as they foster preschool children’s sense of identity and love for their country. Historically, the link between patriotism and the development of citizenship has been of immense importance. Nearly every preschool education program incorporates a patriotic component as part of its overall educational framework for children [7]. Both historical and contemporary researchers have highlighted the significance of this educational focus [8,9]. Many countries have underscored that instilling a sense of patriotism should be a foundational element of education starting in early childhood.

Researchers focus on children’s patriotism to further elucidate its connotations. Closely related to patriotism is the concept of national identity. Barrett et al. systematically elaborated on the content framework of children’s national identity, positing that it, along with patriotism, forms an extremely complex psychological structure comprising two systems: cognition and emotion [10,11]. The cognitive system encompasses children’s awareness of national existence, symbols, customs, traditions, and more. In contrast, the emotional system pertains to children’s subjective sense of belonging to their country, feelings of national pride and shame, and emotional attachment to their homeland, as well as other social emotions. Ashmore et al. argued that collective identity enhances social embeddedness and behavioral participation, in addition to cognition, emotion, and evaluation [12]. In summary, patriotism can be understood as consisting of at least three primary components: cognition, emotion, and behavior.

### 1.2. The Emphasis on Children’s Patriotism in China

In China, patriotism is highly politicized by the government and is extensively promoted within the educational system. Since the establishment of the People’s Republic of China in 1949, the government has advocated for patriotism as an ideological framework that is essential for the nation’s ongoing development and for fostering responsible citizenship [13]. In this context, patriotism is not only a political obligation but also a fundamental quality expected of citizens. Among the core socialist values emphasized in contemporary China, patriotism holds paramount significance. This emphasis arises from the unique characteristics of China’s national context, which include its traditional culture, political culture, and ethnic diversity. In recent decades, patriotic education has become a central focus of the educational agenda in China. The government integrates patriotism into the school curriculum to fulfill political objectives, such as strengthening national unity, suppressing diversity, and cultivating a collective national identity [14].

Chinese education places significant emphasis on fostering patriotism in children at all developmental stages, particularly during their early years. The *Outline for the Implementation of Patriotic Education in the New Era*, issued by the Central Government of the People’s Republic of China, explicitly states that patriotic education should commence in early childhood [15]. The *Guide to the Learning and Development of Children Aged 3–6* is a pivotal policy document in China’s preschool education framework, outlining the developmental characteristics and specific content relevant to children’s growth. This document delineates the social development milestones that children should achieve concerning patriotism. For instance, children aged 3 to 4 should recognize the national flag and anthem, while those aged 4 to 5 should identify themselves as Chinese. Furthermore, children aged 5 to 6 should be aware of some of the nation’s significant achievements [16]. The *Patriotic Education Law of the People’s Republic of China*, which was implemented in 2024, reiterates the importance and necessity of nurturing individual patriotism [17].

### 1.3. The Characteristics and Development of Preschool Children’s Patriotism

Patriotism in children is a matter of global concern. Researchers from various countries have examined children’s patriotism through diverse methodologies. Their findings indicate that individuals begin to develop preferences for their own ethnicity and country at an early age. According to social identity development theory, children can acquire a sense of belonging to a specific group as early as age three, and they start to show a preference for internal groups over external groups by age four [18]. The study of children’s patriotism can be traced back to the 1940s and 1950s. Clark et al. focused on children’s preferences for national images, investigating the choices of black children between white dolls and black dolls [19]. Piaget and Weil conducted research on children in Geneva, arguing that children’s understanding of national territories and regions reflects their comprehension of their country [20].

In recent years, researchers have shown a sustained interest in children’s patriotism. For instance, Hong’s interviews with preschool children suggest that young children possess complex and often surprisingly sophisticated understandings of nationhood and nationality. These understandings are formed by integrating their life experiences with elements of the social discourses prevalent in the larger society [21]. Slotterback’s research examined children’s patriotism through letters they wrote to their teachers during Christmas, revealing that the number of patriotic sentiments and drawings increased in 2001 and 2002 [22]. The question of whether differences exist in children’s patriotism has also garnered the attention of researchers. A study conducted in four multi-ethnic contexts in Croatia, involving primary and secondary school students, found that younger pupils expressed higher levels of ethnic identity and blind patriotism, while constructive patriotism was more frequently articulated by older students. Notably, there were no age differences observed in in-group bias [23].

### 1.4. The Game-Based Assessment

As for the assessment of children’s patriotism, the current research mainly adopts traditional methods such as interview, observation, and questionnaire. For example, Barrett et al. used relatively subjective tasks and attribution tasks to investigate children’s sense of identity and preference for the country [14]. These tasks have fixed test questions and procedures, and are often conducted manually with preschool children. This traditional method has disadvantages such as taking too long and collecting small samples. In addition, during the study, the children were relatively passive to the test and the experience was relatively uninteresting.

Due to the development of human–computer interaction technology, game-based assessment has emerged in the field of child assessment [24]. With the help of human–computer interaction, children can input information through various channels such as voice, gestures, eyes, expressions, etc. Children are exposed to familiar music and cute cartoon characters, and can obtain a richer and more interesting interactive experience [25,26]. Games offer advantages over traditional methods of assessing cognitive function among children and adolescents [27]. For example, people find video games engaging and tend to be more motivated to complete a game-based assessment compared with people performing a paper-and-pencil test [28]. Game-based assessments also ensure fidelity because all participants are interacting with a predefined game, thereby reducing the likelihood of introducing bias by the person scoring their performance [29].

### 1.5. The Present Study

Against the unique social background of China, we studied 3–6-year-old preschool children, with the aim of analyzing their development characteristics relating to patriotism. To conduct the research more intelligently, scientifically, and vividly, the research task was conducted using a game-based assessment tool. Our study of Chinese preschool children was guided by the following four research questions:

(1) What are the characteristics of patriotism development in preschool children in China?
(2) How do Chinese preschool children understand patriotism?
(3) Why do Chinese preschool children love their motherland?
(4) What are the potential types of patriotism among preschool children in China?

## 2. Materials and Methods

### 2.1. Study Design

This study employed a mixed-methods design, integrating both quantitative and qualitative research to obtain comprehensive and in-depth data [30]. The mixed-methods approach is particularly advantageous when there is limited existing research on a topic or when neither quantitative nor qualitative data sufficiently capture the complexity of the phenomenon [31]. Our current research questions, along with the scarcity of related evidence, informed our decision to adopt a hybrid approach. The objective of the quantitative phase was to assess the level and type of patriotism among preschool children. In contrast, the qualitative phase aimed to explore the specific manifestations and underlying causes of patriotism in these children. Throughout the study, quantitative and qualitative methods were interwoven across multiple stages of the inquiry. Ultimately, we juxtaposed and integrated the data from both phases to thoroughly address the question of whether preschool children express love for their home country.

### 2.2. Participants

This study employed stratified convenience sampling to select preschool children aged 3 to 6 years from the provinces of Beijing, Shandong, and Guizhou. The researchers aimed to account for regional diversity as much as possible. Beijing, as the capital of China, represents the northern region; Shandong is a more developed province in the east; and Guizhou serves as a representative of the western region. From the initial convenience selection, one to two cities or regions within each province were chosen through cluster sampling of preschools. A statistical analysis revealed that a total of 1423 preschool children were valid participants, of whom 42.3% were girls. Additionally, 24.4% of the children belonged to the 3–4-year age group (i.e., junior class), 38.8% were in the 4–5-year age group (i.e., middle class), and 36.8% were in the 5–6-year age group (i.e., senior class). The proportions of children from Beijing, Shandong, and Guizhou were 34.8%, 26.9%, and 38.3%, respectively.

### 2.3. Measurement

**Figure 1:**
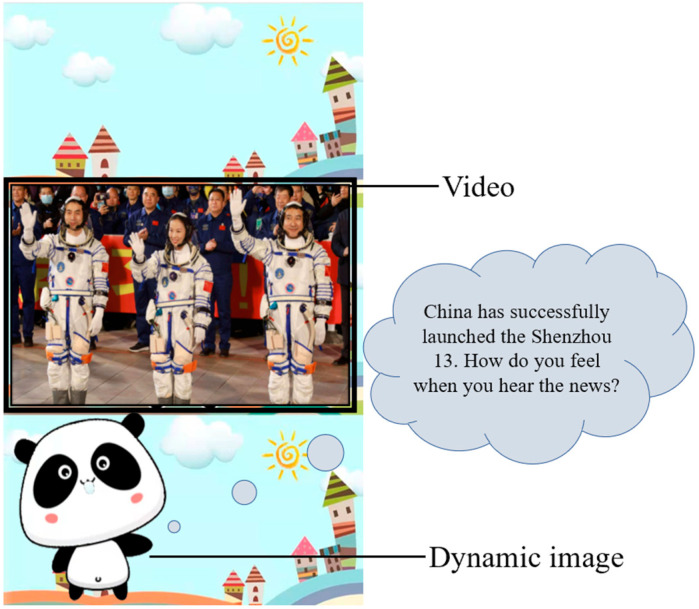
The “Panda Paradise” game.

To encourage preschool children to engage with themes of patriotism, this study utilized the narrative game, “Panda Paradise”, developed by our research team [32]. Given the widespread use of mobile phones and tablet devices, we designed the game specifically for mobile platforms, allowing children to play on their preferred devices. The primary mode of interaction was touch-based, with user engagement facilitated through button clicks, touch screen sliders, gestures, and voice responses, among other methods. To help children grasp the game’s rules, an avatar—a Panda—guided them through the requirements and tasks. Figure 1 shows the details of the game. In the game, the Panda is a dynamic figure that can make movements and communicate with young children through language. In the game, videos or pictures will be used to explain the situation, so as to investigate the patriotism of preschool children through the form of questions. The game unfolds in a running format, enabling children to progress to the next task regardless of whether their previous answer was correct. To sustain the children’s attention, they received encouraging rewards each time they advanced to a new level, with the screen displaying messages such as, “You are doing great! Keep it up”. All qualitative and quantitative data for this study were collected through gameplay and encompassed three key aspects.

#### 2.3.1. Demographic Information

Demographic data comprised a child’s grade, gender (1 = boy, 2 = girl), and home region, all of which were obtained through the game. Given that these children’s written language was limited, we obtained their answers through a verbal question-and-answer process. If a child did not know their age or other information, we obtained those answers from their parents. This part of the information was objective and unique, so parents’ answers did not affect the authenticity of the data.

#### 2.3.2. Assessment of Children’s Patriotism

The game consisted of 31 tasks measuring the three dimensions of national cognitive mastery, national emotion engagement, and national behavior tendency. Each task was designed to measure one indicator of preschool children’s patriotism. National cognitive mastery mainly examines preschool children’s mastery of national knowledge, including 11 items. Each task will be completed by interacting with the Panda. These interactions are situational and not a serious exam. For example, in one play scenario, the Panda asks a child, “Which one is the Chinese flag?” The flags of the four countries will appear on the screen, and preschool children can choose the picture they think is correct. National emotion engagement mainly investigates preschool children’s love for the country, pride, and other emotions, including 11 items. For example, in the context of the Olympic Games, the screen shows Chinese athletes winning the championship. The Panda communicates with preschool children to assess their national pride. National behavior tendency mainly examined preschool children’s patriotic behavior, including nine items, such as participating in national festivals and safeguarding national symbols. For example, in the game scenario of a festival celebration, the Panda will invite preschool children to participate in traditional festivals, and preschool children can choose their favorite festivals and say why. Each item was scored on a scale of 0 to 10. The higher the score, the higher the child’s level of patriotism. This game-based assessment has been used with preschool children in China and has proven to have good reliability and validity [32]. In this study, the Cronbach’s α coefficients of the three dimensions were 0.84, 0.93, and 0.81, respectively.

#### 2.3.3. Interviews with Children about Patriotism

Through the way of interview, we gained a more comprehensive understanding of preschool children’s patriotism, especially their understanding of patriotism and its reasons. The interview was conducted by the game’s virtual character, “Panda”, who plays the role of researcher in traditional interviews. The interview questions were set in advance by the researchers. Taking into account the characteristics of preschool children’s age development, the interview questions are brief. The interview covered three areas: Do you love your country? What does it mean to love your country? Why do you love your country? The system will automatically record the child’s language and transcribe the speech into text through speech recognition. After testing, the tool’s speech recognition accuracy rate is high. Researchers will analyze the text.

### 2.4. Procedures

We selected preschool children aged 3 to 6 through the preschools they attended. After obtaining permission from the preschool, we contacted parents—also through the preschool—and informed them about the study’s purpose and issued informed consent forms. All the parents who were contacted agreed to let their children participate in our study. The preschool teacher forwarded the link to the game through a WeChat group. Children often play games at home using their parents’ phones or tablets, and because of the virtual characters in our game, the children could complete the tasks independently. To avoid the influence of adults, however, we emphasized in the guidelines that the children must complete the game tasks independently. Parents could encourage their children, but they could not tell them the right answer. If the child was interrupted during a game task, they could complete it at another, more convenient time. Information from each response was retained for every log-on as the game progressed. We also collected each child’s age, gender, and other basic information through a game task. To avoid potential bias in societal expectations, we did not collect kindergarten information or the children’s names.

### 2.5. Data Analysis

All the survey data were collected and analyzed using IBM SPSS 24 and Mplus 7.4. First, descriptive analyses, two independent-samples *t*-tests, and a one-way analysis of variance (ANOVA) were performed for the analytic sample. Second, descriptive analyses were undertaken first for the analytic sample. Latent profile analysis (LPA) was then used to identify the latent profiles. LPA is a person-centered approach that uses continuous variables to divide cases into subgroups based on potential similarities [33]. A range of model fit indices was considered to determine the optimal number of profiles. The main indices used included the Akaike information criterion (AIC), Bayesian information criterion (BIC), and sample-size-adjusted BIC (SSA-BIC). Lower values indicated better model fit [34]. Entropy is often used as an index to reflect classification accuracy, with higher values indicating better classification quality. The Lo–Mendell–Rubin (LMR) likelihood ratio test is significant in making a discriminant choice between two models, with k classes against k–1 classes [35,36,37].

The interview transcripts were analyzed qualitatively. First, we used open coding technology to read and encode these data independently. All texts were checked multiple times to find evidence of preschool children’s patriotism (e.g., phrases and sentences) in each specific incident that occurred during the field investigation and coded at the first level. Then, the first level of coding content was classified, that is, the same type of information was put into the same category. Finally, this information was grouped into third-level codes.

## 3. Results

### 3.1. Characteristics of Patriotism in Preschool Children

The descriptive statistics results showed that the mean for the three dimensions of preschool children’s patriotism was 6.93, 7.25, and 6.28, respectively. More specifically, 60.1% of the preschool children reported feeling proud and happy to be Chinese; 70.2% reported feeling proud and happy because China has successfully launched a manned space shuttle. The one-on-one interviews generated more vivid results: 90% of the children said they loved their country. Some children even emphasized their answers, for example, “We should love our own country” and “Children should love their own country”.

MANOVA analysis showed that the interaction between grade and region was significant (*F*_(12,4964)_ = 10.01, *p* < 0.000), the main effect of grade was significant (*F*_(6,3312)_ = 23.67, *p* < 0.000), and the main effect of region was significant (*F*_(6,3312)_ = 8.64, *p* < 0.000). As shown in Table 1, preschool children’s patriotism improved considerably as the grade level increased. The level of patriotism among preschool children in junior classes was significantly lower than that of preschool children in middle and senior classes, and the level of patriotism among preschool children in middle classes was significantly lower than that of preschool children in large classes. However, preschool children’s patriotism only have regional differences in the dimension of national cognitive mastery. Preschool children in the Beijing area scored the highest, significantly higher than those in other non-capital areas. Additionally, the independent samples *t*-test showed no significant gender differences in the preschool children’s scores for all dimensions of patriotism.

### 3.2. Understanding of Patriotism among Preschool Children

Most of the preschool children answered very clearly that they loved their country; however, their understanding of patriotism was vague. In the interviews, some of the younger children simply said they did not know or their answers were unrelated to the question. Most of the preschool children answered their understanding of love for their country. The children explained their understanding of patriotism further by giving examples. It is worth noting that the explanations often referred to national symbols such as the national flag and the flag-raising ceremony. Some of the children believed that loving the national flag and participating in the flag-raising ceremony is loving their country. Following are some representative statements given by the preschool children:

*“Patriotism is to seriously participate in the flag-raising ceremony of the preschool”.*

(Huang)

*“To care for the national flag is to love the country”.*

(Zou)

### 3.3. Reasons for Patriotism in Preschool Children

The study further analyzed the reasons why preschool children love their country. About one in five children did not answer the question or the answer was not clearly related to the question. One in five said, again, “I love my country,” but did not give a specific reason. The remaining three-fifths gave specific answers. Our analysis of these answers found specific reasons for the emergence of patriotism in preschool children. The reasons can be classified into the following four types: emphasizing the achievements of China, emphasizing the good qualities of Chinese people such as kindness, diligence, and bravery, emphasizing the consciousness of national identity, and emphasizing China’s wealth of products (see Table 2).

### 3.4. Potential Profile of Patriotism in Preschool Children

To investigate the potential types of patriotism among preschool children, profile models were established with three indicators: national cognitive mastery, national emotional engagement, and national behavior tendency. Starting from the benchmark model with a class number of 1, we performed the model-fitting evaluation of the potential profiles of preschool children’s patriotism. The potential profile model-fitting index for categories 1 to 5 is shown in Table 3. Values for AIC, BIC, and Entropy indicate that the five class solutions perform slightly better than the others. However, one category of model 5 accounts for only 3%, which is not representative enough. In addition, model 4 has the highest SSA-BIC value, and other indicators are significantly better than other models 2 and 3. The values of the SSA-BIC indicated that the four-class solution performed slightly better than the others. Combining all the indicators, we choose the four-section model as the optimal model for the potential type of patriotism among preschool children. ANOVA was conducted with post-hoc tests to examine whether significant differences existed across the children’s patriotism within each profile. ANOVA was performed using a post-hoc test to examine whether there were significant differences in patriotism among the children in each profile. Post-hoc tests confirmed significant differences in all four of the children’s profiles.

**Figure 2:**
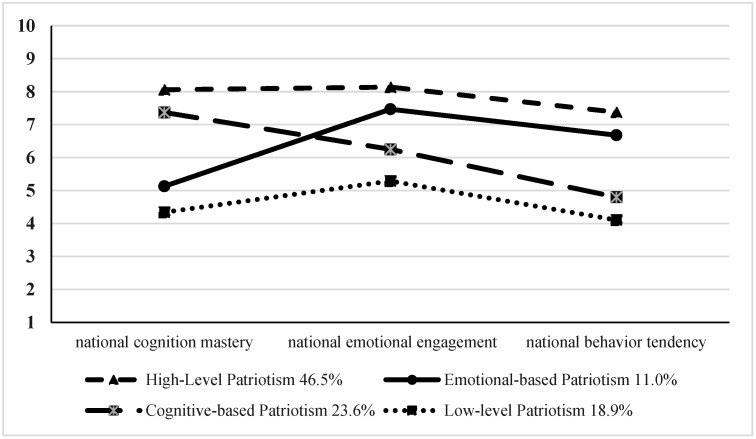
The four profiles of children’s patriotism.

Details of the four profiles for preschool children’s patriotism are as follows (see Figure 2).

Profile 1: High-level patriotism. Children’s patriotism scores were calculated for the three dimensions—national cognitive mastery, national emotional engagement, and national behavior tendency—and ranged from 7.38 to 8.14, demonstrating excellent performance in all three. There were 661 preschool children in this category, accounting for 46.5%.

Profile 2: Cognitive-based patriotism. Children’s patriotism scores for the three dimensions ranged from 4.80 to 7.37. Of the three dimensions in this profile, cognitive mastery scored highest. Furthermore, the scores for national cognitive mastery were significantly lower than those in the first profile and significantly higher than those in other profiles. There were 336 preschool children in this category, accounting for 23.6%.

Profile 3: Emotional-based patriotism. Scores for national cognitive mastery, national emotional engagement, and national behavior tendency were 5.13, 7.57, and 5.88, respectively. Obviously, among the three dimensions, national emotional engagement scored highest, while national cognitive mastery and behavioral tendency scored relatively low. A total of 269 preschool children qualified for this profile, accounting for 11.0%.

Profile 4: Low-level patriotism. Children’s patriotism scores in this profile were less than 5.29. They were characterized by the lowest performance in national cognitive mastery, national emotional engagement, and national behavior tendency, and named accordingly as the low-level group. This group included 18.9% of the preschool children.

## 4. Discussion

Patriotism is an old topic because it concerns the future sustainable development of a country. In the context of globalization, a country pays more attention to its national identity and love. China has always attached great importance to the patriotism of its citizens, including preschool children. This study investigated Chinese preschool children and used game-based assessment to investigate their characteristics and profiles of patriotism. The results showed that patriotism among preschool children was above the average level and increased with age. Preschool children in the capital region scored higher on national cognitive mastery. Preschool children’s understanding of patriotism is vague, and they explain and express their patriotism mostly through concrete examples. The four types of patriotism in preschool children that we identified are high-level patriotism, cognitive-based patriotism, emotional-based patriotism, and low-level patriotism. Our findings will contribute to a more comprehensive understanding of preschool children’s patriotism and supplement the research in this area. More importantly, our results provide an evidence-based reference for patriotic education.

This study mainly used game-based assessment to verify that game-based assessment can be used to assess preschool children’s patriotism. Thanks to technological advantages, the current study process is more interesting, which increases the participation of preschool children. In addition, the large sample was obtained because the technology overcame the limitations of time and space, and preschool children in different regions could complete the game tasks at any time. Since the advantages and effectiveness of this tool have been discussed in previous studies, the use of this tool will not be specifically discussed in this study.

### 4.1. Characteristics of Patriotism among Preschool Children in China

Patriotism includes preschool children’s understanding of, emotions about, and behavior toward their country, which means that children should master certain national knowledge, love their country, and be willing to work hard for their country. The results of this study show that patriotism development among preschool children in China is above a medium level, which is a relatively positive sign. This may have something to do with the efforts of the Chinese government and educational institutions. As mentioned above, important policy documents in the field of preschool education, such as the “Guide to Learning and Development for Children Aged 3–6”, emphasize the need for preschool children to have a sense of national identity [16]. At the national level, China attaches great importance to patriotic education for preschool children [38,39]. Furthermore, preschools will actively carry out educational activities in patriotic education. In the modern world, festivals and commemorative ceremonies, national pledges, and songs are all used to help students connect to each other and their wider society, particularly to the nation-state and its ideals [40]. For example, in China, preschools hold a flag-raising ceremony every Monday; raising the flag is a very ceremonial event, including singing the national anthem, raising the national flag, and speaking under the national flag. In such a solemn event, preschool children can recognize the national flag, become familiar with the national anthem, and learn to respect the flag (that is, look at the flag with their eyes and refrain from whispering when the flag is being raised). Additionally, preschools organize theme activities during National Day to celebrate the birth of the motherland through singing, painting, and other activities.

A comparison of the three dimensions of patriotism found that the score for national emotional engagement was the highest and the score for national behavioral tendency was the lowest, which indicates that preschool children have better patriotic emotional development, but poorer behavior. This is also in line with the developmental characteristics of preschool children. Preschool children are in a stage of physical and mental development, in which national behavior tendency is weak because of their lack of cognitive ability, self-control, and mature behavioral habits. Another finding of this study that can help explain this problem is that preschool children’s understanding of patriotism is very vague. For adults, loving their motherland means not only emotional involvement, but also behavioral involvement [2], as Chinese Premier Zhou Enlai once said, “to study for the rise of China”. Patriotism certainly includes belief and affection for one’s country, and the litmus test of patriotism is what one is prepared to do for their country. Similarly, Ladson-Billings argues, “Patriotism is not what you say; patriotism is what you do [41]”.

### 4.2. Differences in Patriotism among Preschool Children in China

Our research showed age differences in patriotism among preschool children. As the children’s age increased, the level of patriotism improved. This result accords with the law of children’s physical and mental development: as children grow older, their cognitive abilities and behavioral norms typically improve. Furthermore, with the increase in patriotic education, their level of patriotism will also increase. Indeed, this finding is consistent with previous research by Barrett et al., who found that with an increase in age, children’s in-group prejudice against ethnic groups generally declined [42].

In addition, our findings found that children in the metropolitan area performed best in national cognitive mastery. Previous studies have found that children in the capital region show higher levels of patriotism and national identity. For example, a study by Riazanova et al. found that children living in the capital rated national identity as more important than children living in other areas [43]. Barrett et al. found in the study of British children that English children’s subjective importance of being British and their sense of identity with British were significantly stronger than Scottish children [44]. We believe that preschool children living in Beijing have more opportunities to know national symbols and contact more national activities, and thus obtain a higher level of national cognitive mastery score. For example, Beijing has the Tiananmen Square, the Great Wall, the Forbidden City, the National Museum, and other famous buildings, which contain a lot of national symbols, traditions, achievements, and so on.

Our findings indicated that although children in this age range emphasize that they love their country, they do not fully and accurately understand what patriotism represents. This is related to the cognitive development of preschool children. It is clear from the preschool children’s answers that their understanding of patriotism is usually combined with specific examples. Children at this stage engage in concrete image thinking; they respond to specific events, and examples can help them better understand and express their feelings and ideas.

### 4.3. Profiles of Patriotism among Preschool Children in China

A person-centered approach has earned much attention because it more closely approximates practice and is easier to understand. LPA is one analytical tool typically used for such analysis [45,46]. We used this method to examine the types of patriotism among preschool children and observed heterogeneity in their patriotism, which we categorized into four types. Of these, the low-level patriotism type accounted for a relatively low proportion, indicating that although there are differences in patriotism among preschool children, the number of children with a low overall level of patriotism is small. Thus, it can be inferred that preschool children’s patriotism development as a whole is in a relatively good state in China. This is also essentially consistent with the results of this study for the characteristics of preschool children’s patriotism.

Finally, the diversity of patriotism types among preschool children has inspired educators to adopt different education programs according to different characteristics of children’s patriotism. While the number of preschool children with low levels of patriotism is small, this type of child cannot be ignored in practice. Educators should accurately identify this type of child and enhance their patriotism in a variety of ways.

## 5. Limitations

The limitations of the current study and potential avenues for future research should be considered. First, although data for three regions were selected for analysis, China is vast, and therefore future studies should consider more representative samples, taking into account both urban and rural areas. Second, the background information we collected about participants was only basic, which limits further analysis of the data. Third, this study adopted a new assessment method—game-based assessment—which has many advantages, such as greater involvement of children and more objective data. Future research can improve the design using game-based assessment, and other methods can be used to collect demographic background variables to obtain more complex relationships between the data, while ensuring its validity. Fourth, some necessary research needs to be carried out. To determine whether children, especially younger children, understand what a country is, they should first be interviewed about their understanding of the country, and then whether they love their country and why. Fifth, although researchers emphasize that parents should let their children complete the game tasks independently, there is no way to prevent parents from helping their children. Parental guidance may influence a child’s response. Sixth, some necessary influencing factors, such as parents’ patriotism and previous patriotic education, can be taken into account in future studies. Seventh, future research is necessary to more fully and deeply understand the differences between the game-based assessment and traditional assessment. For example, whether the game itself affects children’s expressions of patriotism

## 6. Practical Implications

This study has important practical significance for improving preschool children’s patriotism, and can provide a reference for teachers, parents, and policy makers. First, some specific activities may help to increase patriotism among preschool children. Whether in kindergartens or families, activities related to national culture and traditional festivals can be organized so that preschool children can understand their own country through personal participation and thus have patriotism stimulated. Second, the content of patriotism education should be determined according to each age group. This study found significant differences in the level of children’s patriotism at each age, and thus their educational content should also differ. Third, it is necessary to pay attention to children with low levels of patriotism. Teachers and parents must take measures to improve the patriotism of such children. For example, parents can let their children learn more about Chinese culture, history, and modern achievements by reading picture books, traveling, and watching cartoons, and discuss things related to China with their children in simple words.

## Figures and Tables

**Table 1:** Means and standard deviations in patriotism of preschool children in different grades and regions.

	National Cognitive Mastery	National Emotion Engagement	National Behavior Tendency
Junior class	6.40 (2.27)	6.82 (2.02)	5.54 (2.75)
Middle class	6.45 (2.35)	6.93 (2.12)	6.20 (2.67)
Senior class	7.80 (2.24)	7.89 (1.95)	6.92 (2.39)
*F*	51.05 ***	30.90 ***	19.84 ***
Beijing	8.22 (1.50)	7.64 (1.77)	6.40 (2.41)
Shandong	6.72 (2.65)	7.35 (2.15)	6.15 (2.61)
Guizhou	7.07 (2.12)	7.10 (2.07)	6.30 (2.73)
*F*	12.92 ***	2.34	1.99

Note. *** *p* < 0.001.

**Table 2:** Preschool children’s reported reasons for pride in their country.

Reasons	Specific Expression
Emphasize China’s achievements	“Because of internal security, we live every day in peace and no one beats us”. “Because we beat the Japanese”. “Because China can get free vaccines”. “Now the new coronavirus is here, and our doctors have chased the virus away during the day, so I want to thank China, thank us for the day”. “Emphasizing the good qualities of Chinese people”.
Emphasize the good qualities of the Chinese people	“Chinese people are very kind”. “Chinese people are hardworking and brave”. “The Chinese have done some particularly great things in history”.
Emphasize the consciousness of national identity	“Because the motherland is my home, we must love the motherland”. “I’m Chinese, so I have to be proud”.
Emphasize that China is rich in natural products	“The places in China are beautiful and nice”. “Because there’s good food around and you can play”.

**Table 3:** Model fit indicators for the latent profiles of children’s patriotism.

Model	AIC	BIC	SSA-BIC	Entropy	LMRT (*P*)
1	44,556.60	44,600.95	-	-	1
2	27,438.18	27,512.11	0.87	16,682.39 ***	0.37, 0.63
3	20,818.33	20,921.83	0.89	6456.01 ***	0.08, 0.37, 0.55
4	18,795.59	18,928.67	0.91	1978.08 ***	0.46, 0.11, 0.24, 0.19
5	17,994.46	18,157.10	0.83	788.15 ***	0.08, 0.03, 0.22, 0.25, 0.42

Note. *** *p* < 0.001; AIC = Akaike information criterion, BIC = Bayesian information criterion, SSA-BIC = Sample-size adjusted BIC, LMRT = Lo–Mendell–Rubin test.

## Data Availability

The data presented in this study are available on request from the corresponding author.
